# Research Advances in Marine Aquaculture Net-Cleaning Robots

**DOI:** 10.3390/s24237555

**Published:** 2024-11-26

**Authors:** Heng Liu, Chuhua Jiang, Junhua Chen, Hao Li, Yongqi Chen

**Affiliations:** 1 College of Science and Technology Ningbo University, Ningbo 315300, China; 2 College of Science and Technology, Ningbo University, Ningbo 315211, China

**Keywords:** marine culture, net cleaning, cleaning robot, underwater image acquisition, motion control

## Abstract

In the realm of marine aquaculture, the netting of cages frequently accumulates marine fouling, which impedes water circulation and poses safety hazards. Traditional manual cleaning methods are marked by inefficiency, high labor demands, substantial costs, and considerable environmental degradation. This paper initially presents the current utilization of net-cleaning robots in the cleaning, underwater inspection, and monitoring of aquaculture cages, highlighting their benefits in enhancing operational efficiency and minimizing costs. Subsequently, it reviews key technologies such as underwater image acquisition, visual recognition, adhesion-based movement, efficient fouling removal, motion control, and positioning navigation. Ultimately, it anticipates the future trajectory of net-cleaning robots, emphasizing their potential for intelligence and sustainability, which could drive the marine aquaculture industry towards a more efficient and eco-friendly era.

## 1. Introduction

In recent years, marine aquaculture has gained significant attention as a crucial method of meeting global seafood demand. According to the Food and Agriculture Organization (FAO) of the United Nations’ 2022 statistical report [1], marine aquaculture contributes substantially to the total value of global fisheries and has emerged as a primary driver of economic development in the fishing industry. However, the expansion of aquaculture on such a scale also brings with it numerous challenges.

Cage systems are essential infrastructure in marine aquaculture. However, during the cultivation process, the netting accumulates significant debris and biological fouling, which can hinder water exchange and compromise the safety of aquaculture operations [2,3,4]. To tackle this issue, various cleaning methods have been explored. In the early stages, manual salvage cleaning was the primary method used. Although it was simple to operate, its high labor intensity and low efficiency gradually revealed several drawbacks [5]. By the end of the 20th century, with the advancement of mechanical equipment, mechanical flushing systems began to be applied to net box cleaning. These systems use high-pressure water flow to remove contaminants, significantly improving cleaning efficiency [6]. However, mechanical cleaning requires advanced technical skills and may negatively affect both the materials of the net boxes and the aquaculture environment.

To further enhance efficiency and minimize environmental impacts, environmentally friendly cleaning methods have been developed in recent years, such as the use of antifouling coatings and biofouling techniques [7,8]. Antifouling paint reduces biofouling through a chemical coating, but there is a potential risk of chemical contamination [9]. On the other hand, biofouling control employs a proportional mix of cleaner fish to reduce adhesion buildup. Despite its environmental advantages, this method has a limited scope of application, and its effectiveness is constrained by the behavioral characteristics of the fish [10].

With the rapid development of artificial intelligence and robotics, intelligent net-cleaning robots have gradually become a research hotspot. Due to their flexibility, high efficiency, and environmental adaptability, these robots are capable of performing precise net-cleaning tasks in varying marine environments [11]. However, existing methods involve trade-offs between environmental adaptability, cleaning efficiency, and practical application costs, and have not yet resulted in a widely applicable solution. In particular, systematic research on the integration of efficient cleaning equipment and intelligent technology is notably lacking. Furthermore, while existing research mainly focuses on individual cleaning technologies, there is insufficient discussion on the integration, intelligence, and sustainable development of net-cleaning technologies.

In view of this, this paper focuses on the application and key technologies of net-cleaning robots in marine aquaculture, aiming to fill the research gaps in this field. First, we systematically review the current status of net-cleaning robot applications, analyzing typical use cases in aquaculture net cleaning and underwater detection. We then discuss the development status and challenges of the five core technologies involved in net-cleaning robots, including visual recognition, net sticking movement, high-efficiency decontamination, motion control, and underwater localization and navigation. Finally, we examine the future directions of integrating and intellectualizing these key technologies, providing both theoretical support and practical guidance to advance technological progress and promote the practical application of this field.

The structure of this paper is as follows: Section 2 summarizes the current status of net-cleaning robot applications in marine aquaculture; Section 3 provides a detailed analysis of the research progress and challenges of key technologies; Section 4 offers an outlook on future development trends and concludes with a summary of the entire paper.

## 2. The Current Status of Application for Net-Cleaning Robots in Aquaculture

The application of net-cleaning robots in marine aquaculture has emerged as a pivotal strategy for enhancing production efficiency, refining management practices, and safeguarding marine environments. This section explores the current status of net-cleaning robots, covering their roles in net cleaning, underwater inspection, and monitoring in marine aquaculture. These applications not only improve cultivation efficiency but also substantially reduce costs and decrease reliance on human labor.

### 2.1. Net Cleaning in Aquaculture

The net-cleaning robot efficiently performs the underwater cleaning of nets by integrating a control system, a motion module, and a cleaning unit. The control system is designed to flexibly adjust cleaning speed and duration based on the type and distribution of adherents on the nets, enabling precise and targeted operations [12]. The cleaning unit incorporates advanced tools, such as water jet nozzles and brushes, which effectively eliminate debris, dirt, and biological fouling through high-pressure water jets or mechanical brushing [13]. This ensures both the efficiency and reliability of the cleaning process.

**Figure 1:**
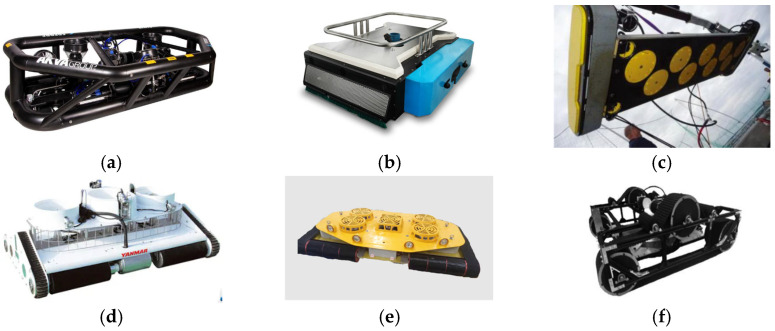
Overview of net-cleaning robots. (**a**) Flying Net Cleaner 8 net-cleaning robot; (**b**) Mainstay-Weda cleaning robot; (**c**) Aurora Marined net-cleaning robot; (**d**) Yanmar Marine net-cleaning robot; (**e**) Banlan Marine net-cleaning robot; (**f**) Net-cleaning robot of Guangdong Ocean University [14].

The Flying Net Cleaner 8 (FNC8), designed by the Norwegian company AKVA, enables the entire net-cleaning process to be controlled from a centralized control room. Unlike traditional methods using wheels or belts, the FNC8 crawls along the cages, minimizing wear and tear on the nets. Another underwater robot, jointly developed by Swedish companies WEDA Water and Mainstay is specialized in removing algae and sediment deposits from vertical or horizontal surfaces in aquaculture settings. Aurora Marine, based in the UK, has engineered a track-driven underwater net-cleaning robot that ensures stable adhesion to cage surfaces, equipped with a high-pressure water jet system for effective netting cleaning. Yanmar Marine, a Japanese firm, has introduced a net-cleaning robot employing a unique method combining high-pressure water jets with brush scrubbing. This hybrid cleaning approach significantly boosts efficiency and effectively removes persistent dirt and biological fouling from cages. China’s Colorful Ocean Technology Co., Ltd. has developed an automated net-cleaning robot that attaches to nets in need of cleaning through a thrust-adhesion mechanism. The robot can be manually controlled or operated autonomously, moving across the nets while utilizing cavitation jet technology to remove debris from the nets. Meanwhile, Liu Siyuan and colleagues from Guangdong Ocean University [14] have developed a fully automated deep-water net-cleaning robot. This robot also employs cavitation jet technology for cleaning and uses a track system to traverse the nets. Additionally, it is equipped with an underwater camera for visual monitoring of the surroundings. Figure 1 is an overview of the net cleaning robot.

Despite significant advancements in net-tank cleaning robotics in recent years, accompanied by a growing body of related technologies and research, several fundamental challenges remain unresolved. Firstly, the evolving aquaculture environment, particularly the demands of cleaning deep-water and structurally complex nets, poses challenges that current technologies have yet to effectively address. Existing cleaning robots often exhibit limited adaptability and flexibility when handling various net configurations, such as irregularly shaped nets or three-dimensional structures, thereby restricting their application in complex aquatic conditions [15]. Secondly, a notable trade-off exists between cleaning efficiency and equipment durability of current cleaning technologies. While high-pressure water jet technology is highly effective in removing adherents on nets, its intense impact can lead to abrasion of the netting, potentially compromising the structural integrity and lifespan of the nets over extended periods of use [16]. In addition, while existing automated cleaning systems have enhanced cleaning efficiency to some extent, their level of automation and adaptability remain inadequate. This is particularly evident in complex water flow environments and deep-water conditions, where the stability and intelligence of automated systems falls short of ideal standards, thereby hindering their widespread adoption in practical applications [17].

### 2.2. Underwater Detection and Monitoring

In the field of marine aquaculture, maintaining stable water quality, net integrity, feed distribution, and the health of fish populations is crucial. Traditional detection and monitoring methods, which primarily rely on manual observation or stationary sensor equipment, often result in limited monitoring coverage, slow data update rates, and insufficient information. Net-cleaning robots can be equipped with various advanced sensors, such as water quality monitors, imaging cameras, and sonar systems. These devices enable real-time data collection and analysis of the aquaculture environment and facilitate the rapid transmission of information to the onshore control center, ensuring timely updates of monitoring data.

**Figure 2:**
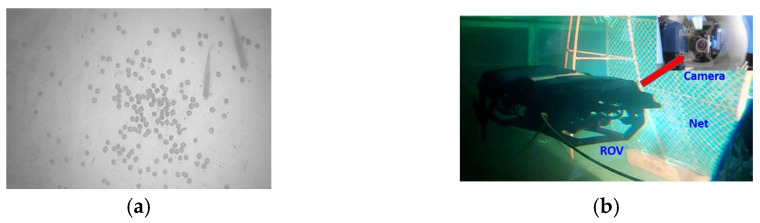
Seawater net-pen aquaculture monitoring scenarios. (**a**) Surplus bait monitoring [20]; (**b**) Fishing net breakage monitoring [21].

To effectively monitor and mitigate potential negative impacts in marine aquaculture, Karimanzira [18] proposed a navigation method that combines behavior-based control with waypoint tracking, equipping Autonomous Underwater Vehicles (AUVs) with sensors, such as conductivity and dissolved oxygen monitors to facilitate large-area water quality monitoring. Researchers at the Norwegian University of Science and Technology (NTNU), including Osen [19], developed a Remotely Operated Vehicle (ROV) that uses small propellers for multi-degree-of-freedom movement, enabling multi-factor perception in shallow water aquaculture environments. As shown in Figure 2, Seawater net-pen aquaculture monitoring scenarios. Li [20] employed an adaptive threshold image-processing method to automate feed monitoring in aquaculture under varying water turbidity and uneven lighting conditions. Additionally, Dalian University of Technology [21] integrated an advanced image acquisition system on underwater robots, using adaptive image-processing technology to achieve automatic recognition and the precise localization of damage to marine aquaculture net cages.

Despite the significant potential advantages of net-cleaning robots in marine aquaculture, the practical application of current technology still faces several challenges. First, many existing net-cleaning robots on the market are bulky, limiting their operational flexibility in confined or complex net-cage structures and reducing their applicability in diverse aquaculture environments. Second, although these robots are designed to improve cleaning efficiency, their performance often falls short of expectations in practical applications. Additionally, some net-cleaning robots are equipped with high-energy-consuming devices, resulting in short operational times and an inability to meet the demands of large-scale or long-term cleaning operations. Furthermore, the complexity of the underwater environment can interfere with signal transmission, leading to unstable communication and affecting the real-time control and data transmission of the robots.

To overcome these challenges, future research and development efforts should focus on the following areas: First, by utilizing lightweight materials and optimizing designs, robot size can be reduced, thereby enhancing operational flexibility in complex net-cage structures. Second, refining energy management and cleaning technologies—such as developing energy recovery systems or adopting energy-efficient cleaning methods—can extend robot working time to meet the demands of long-term operations. Lastly, the development of more advanced underwater communication and control technologies, such as multimodal communication systems and adaptive control algorithms, should be prioritized to improve the stability and reliability of robots in complex underwater environments.

The application of net-cleaning robots in marine aquaculture not only significantly enhances cleaning efficiency and farming management precision but also effectively reduces costs and safety risks by decreasing reliance on manual labor. As technology continues to advance, the automation and intelligence of net-cleaning robots are expected to improve further. It is anticipated that these robots will expand into additional areas in the future, driving the marine aquaculture industry towards greater efficiency and environmental sustainability.

## 3. Core Technology of Net-Cleaning Robot

Although underwater robotics has found widespread application in various fields, such as underwater exploration, rescue, and structural maintenance, research specifically focused on net-cleaning robots has not yet garnered sufficient attention. The existing literature primarily addresses generic underwater robot technologies (e.g., underwater image recognition, localization and navigation, and motion control) and does not adequately consider the unique challenges associated with net cleaning. Net-cleaning robots must not only possess the localization and motion control capabilities required of traditional underwater robots but also address the specific needs for precise net cleaning in complex underwater environments, particularly in terms of efficient decontamination and net-adhering motion. Therefore, this section will examine the advancements in net-cleaning robots across the following core technological areas: underwater image acquisition and visual recognition, net-adhering motion technology, high-efficiency decontamination technology, motion control technology, and underwater localization, navigation, and motion-planning technology. The focus will be on analyzing their applications and technological innovations in the context of net-cleaning tasks, aiming to provide both technical support and a theoretical foundation for the future development of aquaculture automation.

### 3.1. Underwater Image Acquisition and Visual Recognition Technology

**Figure 3:**
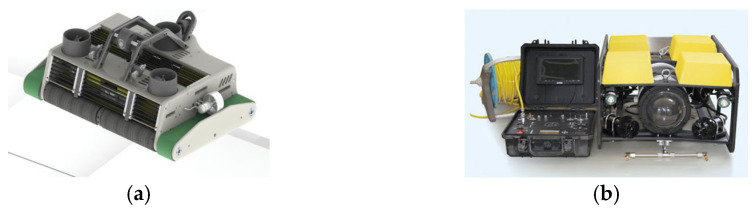
Underwater vision technology for net-cleaning robots. (**a**) Sencott net-cleaning robot with underwater HD camera; (**b**) Integrated Underwater Sonar for Net Washing Robots at the Institute of Automation, Shandong Academy of Sciences, China [22].

When performing cleaning tasks, the net-cleaning robot can capture images using the onboard underwater high-definition camera and sonar system. The underwater HD camera provides high-resolution images of the aquatic environment, while the sonar system offers distance and shape data. This enables the robot to effectively identify fouling, biological attachment, and potential structural damage during operation. Figure 3 shows the underwater vision technology of the cleaning net robot. The net-cleaning robot developed by Qingdao, China Sencott Technology Co., Ltd. in China employs cavitation jet technology to clean net coatings. It is equipped with an underwater HD camera and lighting systems, allowing the operators in the control room to observe the underwater environment in real time. The Institute of Automation at the Shandong Academy of Sciences [22] developed an automated cleaning robot for cavitation jet nets. It integrates technologies such as underwater sensor systems, sonar detection, and embedded control, enabling unmanned and automated underwater net-cleaning operations in high-pressure, anoxic, and low-visibility environments. The use of these technologies not only enhances the robot’s performance but also provides robust technical support for the automation and intelligence of underwater operations.

Underwater recognition technology for net-cleaning robots can be classified into two types: optical recognition and acoustic recognition technology [23]. Optical recognition involves using image data captured by underwater high-definition cameras, which are then analyzed through image processing algorithms to extract feature information. This allows the robot to identify and classify underwater objects. In the field of research in optical recognition technology, Forst [24] designed a remotely maneuverable underwater robot (ROV) for aquaculture, which enables fish identification and data collection by carrying a high-definition video camera. Furthermore, Jinquan Zhang [25] developed a frame-type AUV equipped with a video system and proposed a strategy for detecting spiral net coat breakage. They also developed the corresponding three-degree-of-freedom AUV control system hardware and software through motion modeling and dynamic computations. This research achieved the automated detection of net coat breakage in offshore nets, significantly improving the maintenance efficiency and safety of net aquaculture and reducing the risks and costs associated with manual inspection.

In net-pen farming areas, the light scattering, absorption, and object reflection characteristics of the underwater environment can cause a blurring of images and information loss. This not only affects the clarity of the images but also significantly reduces the accuracy and reliability of target-detection algorithms. To address this issue, underwater image enhancement has become a crucial pre-processing step to improve the performance of recognition techniques. By increasing image contrast, reducing noise, and enhancing edge details, these techniques can significantly boost the visual recognition capabilities of net-cleaning robots, while also improving cleaning efficiency and optimizing net-cleaning accuracy.

To address the issue of image distortion caused by underwater debris, net attachments, and other factors, Petit [26] proposes a quaternion-based attenuation inversion method to address image distortion caused by underwater fouling and net attachments. The method first applies quaternion-based color space contraction and then enhances the image using the principle of light attenuation inversion. Liang [27] improved the transmission estimation of the underwater dark channel prior (UDCP) by incorporating visual factors such as wavelength-dependent attenuation caused by mesh coat attachments and fouling, as well as image blurriness relative to luminance, thereby further enhancing the image clarity and contrast. Liu [28] designed a cascaded multi-module image enhancement framework, which includes image enhancement modules with an adaptive color channel compensation network (AC3Net) and effectively mitigates the underwater-specific color distortion issue problem through simulation experiments.

In the field of underwater image enhancement, deep-learning techniques can automatically learn and extract features from the complex underwater environment, significantly improving the accuracy and efficiency of image processing. Han [29] reviews various intelligent de-fogging and color restoration algorithms, highlighting the great potential of deep learning techniques in solving absorption, scattering, and color distortion problems specific to underwater images. To address the issues of color deviation and detail blurring caused by dirt, which are common in underwater images, Zhou [30] developed a deep learning-based model and fused pixel-weighted channel attention module (PCAM) to enhance the model’s ability to recognize and process underwater image features. Siqi Lu [31] proposed a two-phase underwater image enhancement method based on convolutional neural networks. This method involves a two-phase processing flow, including image corruption and restoration, to enhance the visual quality of the image. It employs convolutional neural networks to address common underwater image issues such as color distortion and poor clarity. Yuzhen Liu [32] proposed an underwater image-enhancement method based on multiscale feature fusion and an attention network, which constructs feature channels and pixel attention residual blocks (CPARs) to achieve dynamic modulation of multilevel features. This approach effectively enhances image details while suppressing redundant information.

Although deep learning has achieved some progress in the field of image enhancement, it still faces challenges in net coat cleaning tasks due to the limited datasets of net coat attachments, which constrain the training and generalization capabilities of deep learning models. To address the issue of insufficient underwater training image data, Chen [33] proposed a perceptual underwater image enhancement method that integrates deep learning with physical priors and introduces the HybridDetectionGAN network framework. This framework fuses physical models and data-driven cues to synthesize training data, and incorporates detector interaction optimization to enhance the model’s adaptability to real underwater images.

Acoustic identification, on the other hand, relies on sonar systems, which emit and receive sound waves, to detect objects. When sound waves encounter an object, they produce echoes, and by analyzing the travel time and intensity of these echoes, sonar can determine the object’s distance, size, and shape. However, when sonar signals propagate in the underwater environment, factors such as water currents and seafloor topography interact along the sound wave propagation path, leading to signal distortion and noise generation. The complexity of sonar signals and the interference from noise significantly increase the difficulty of sonar data processing and identification. In Sonar Signal Processing Research, Wang [34] applied the golden section ratio to the data denoising process of non-local spatial information in their sonar data denoising study to adaptively adjust the filtering degree parameter. This parameter was used to optimize the performance of the denoising algorithm and effectively suppress noise in underwater sonar images. Li [35] applied a non-local low-rank algorithm to denoise underwater sonar images by exploiting the inherent laminar structure information. They enhanced the orientation image as a bootstrap image and calculated robust bootstrap weights based on this image for accurate similar block selection and noise removal. Cheng [36] combined the self-supervised denoising technique with a dynamic attention mechanism to propose a self-supervised blind-spot network denoising structure based on a global awareness strategy. This strategy utilizes a mask-proposal-guided perceptual loss to enhance the model’s adaptability to sonar image characteristics. Table 1 systematically summarizes the research progress of underwater optical and acoustic recognition technology.

For net-cleaning robots in underwater operations, optical recognition provides high-resolution images at close range, making it suitable for fine detection, while acoustic recognition is more effective for long-distance detection. When detecting dirt and damage to the netting, optical recognition is more practical due to its ability to capture fine details. At the same time, acoustic recognition is essential for environmental perception and obstacle detection. With the continuous advancement of sensing technology, the integration of multimodal sensing systems will further enhance the underwater operational capabilities of the net-cleaning robot and advance the development of underwater automation and intelligence to a deeper level.

### 3.2. Net-Cleaning Robot Sticking to the Net Movement Technology

Marine net cage culture is an important part of marine resource development. Since most current sea ranch nets are made of flexible or semi-flexible materials [37,38], the mobile mode of the net-cleaning robot must be adapted to these characteristics in order to achieve effective cleaning operations. Currently, the three main modes of movement for net-cleaning robots include wheeled, footed, and tracked [39]. As shown in Table 2, wheeled mobility is easy to operate, but its limited ability to cross obstacles on the mesh coat and its susceptibility to entanglement limit its applicability to complex mesh surfaces [40]. Although foot-type mobility offers high flexibility, it struggles to meet the demands of large-scale cleaning operations due to its small contact area, slow speed, and limited load capacity [41]. In contrast, tracked mobility, with its larger contact area and higher friction, demonstrates superior obstacle-crossing capabilities and stability [42], making it particularly well-suited for moving across the surface of a mesh coat.

**Figure 4:**
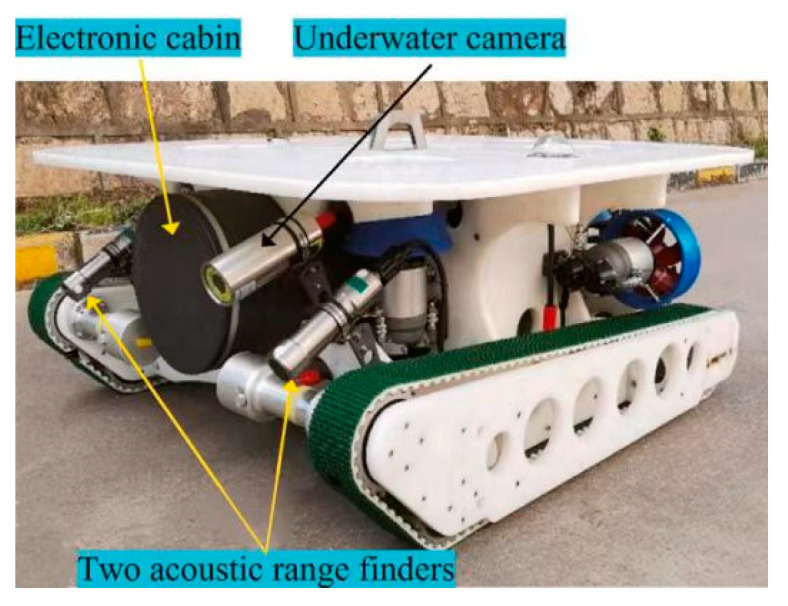
Northwestern Polytechnical University Tracked Hull Cleaning Robot [43].

The School of Marine Science and Technology, Northwestern Polytechnical University [43], developed an underwater robot for hull-cleaning tasks (Figure 4). This robot is equipped with six propellers and two crawler traveling mechanisms. A coherent sliding-mode controller was employed to effectively address synchronization issues between the crawler mechanisms and inaccuracies in depth measurement caused by changes in the underwater environment. Guangdong Ocean University [44] designed a triangular tracked wheel system as a mobility system for a net-cleaning robot. This design combines the flexibility of tires with the stability of tracks, significantly enhancing the robot’s mobility and adaptability in complex underwater conditions.

**Figure 5:**
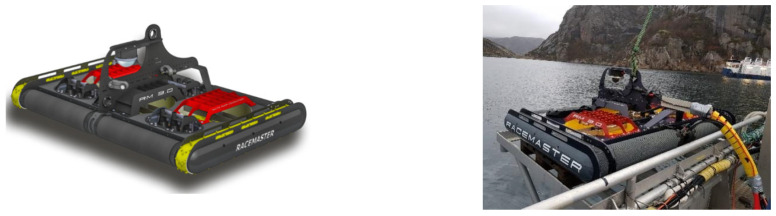
Racemaster net-cleaning robot with tracked movement and integrated adaptive gripper mechanism.

The cleaning efficiency of a net-cleaning robot is closely related to its adhesion force to the net coat. To ensure stable attachment to the net coat under various ocean conditions, an adaptive grappling hook mechanism can be integrated into the crawler traveling mechanism, in addition to optimizing its mobility system, to enhance the robot’s attachment force. Multi Pump Innovation AS has developed the Racemaster series of net-cleaning robots with tracked mobility and an integrated adaptive grappling hook mechanism. This design enables them to handle heavy workloads and maintain stable cleaning efficiency even in conditions with high current speeds or loose netting. Figure 5 shows the Racemaster net cleaning robot. The Chinese Academy of Fisheries Research [45] developed a crawler-type mesh coat cleaning robot for seawater net tank aquaculture. The outer surface of the crawler is equipped with specially designed gripping teeth, which can be precisely embedded in the mesh of the net coat, ensuring a close fit between the robot and the mesh coat for stable gripping.

**Figure 6:**
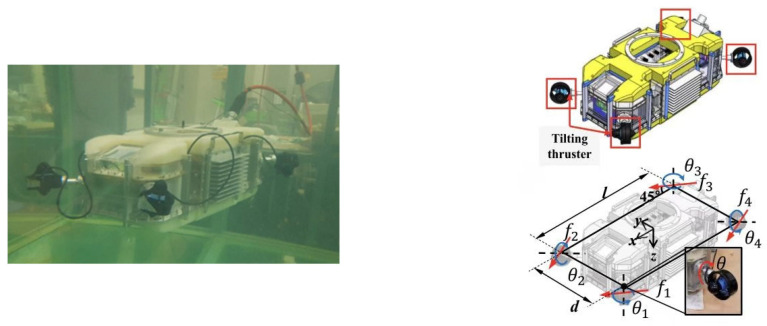
Six-degree-of-freedom hover-controlled tilt thruster and thrust configuration for underwater robots [49].

To ensure that net-cleaning robots maintain mobility and agility in complex and dynamic underwater environments, they are often equipped with screw propellers to enhance their 3D spatial maneuverability. However, since the helical thruster is a nonlinear inertial link, uncoordinated thrust control among multiple thrusters during operation may lead to phase differences between the thrusters, which in turn affects the stability and maneuvering accuracy of the robot [46]. Fossen [47] provides an in-depth analysis of various thrust allocation methods for overdrive vehicles and emphasize that the output saturation constraints of the thruster and limitations on the thrust rise rate must be considered. On this basis, Akmal [48] proposed a fault adaptation strategy based on the Takagi-Sugeno (T-S) fuzzy method to address the active fault-tolerant control problem in underwater vehicle propulsion systems. This strategy maintains the normal operation of underwater robots by dynamically adjusting the control approach in the event of propulsion failures, thereby improving the robustness and safety of the system. Bak [49] employed the decomposition method to address the nonlinearity of the thrust vector caused by the tilting mechanism. He applied the zero-space projection technique to minimize the thrust and avoid reaching the boundaries of the tilting angle, successfully solving the thrust distribution control problem through simulation experiments, as shown in Figure 6. Wang [50] addressed the layout and control allocation problem of the spherical magnetically coupled thruster for underwater robots (SRMCT-II) by using a genetic algorithm to determine the optimal thruster layout independently of traditional engineering experience. They solved the control allocation problem using the augmented Lagrangian method.

The net-cleaning robot integrates multiple technologies by combining an adaptive grappling hook with a tracked mobility system, along with an intelligent control system and thruster technology. This not only significantly enhances the adhesion force between the robot and the net coat but also improves the efficiency and safety of its cleaning operations in a dynamic marine environment. Moreover, this technological advancement supports the sustainable development of mariculture and environmental protection, paving the way for a more efficient and environmentally friendly approach to the future utilization of marine resources.

### 3.3. Efficient Decontamination Technology for Net-Cleaning Robots

Net coating decontamination equipment for net-cleaning robots typically integrates brush and water jet technologies [51]. During the cleaning process, the brushes remove algae, microorganisms, and other organic pollutants from the net coating through physical contact. Water jet technology, known for its high efficiency and energy-saving features, rinses away loose dirt and ensures the thorough cleaning of the net coat. With technological advancements, net-cleaning robot decontamination can be further categorized into lightweight scraping and brushing technology and high-energy consumption water jet technology. Table 3 compares the characteristics and applications of mesh decontamination technology.

Lightweight Scrubbing and Flushing Technology enables efficient cleaning of the net coating with low energy consumption by integrating scrubbing and low-pressure rinsing functions. It ensures no damage to the mesh material while effectively removing algae, microorganisms, and other organic contaminants. Wei Song [52] designed an underwater net-cleaning robot that incorporates a negative suction component, a support member, and a rotatable scraping device. During the cleaning process, the scraping device effectively scrapes the net coat while the negative pressure suction system operates synchronously to instantly remove the scraped debris. Zhu Zhengkai [53] designed a net-cleaning device that integrates a traveling assembly with two sets of scraping assemblies. It is equipped with a rotating rod with an impeller and a spiral scraper with a collection hopper. This device accurately scrapes dirt from the net while moving and simultaneously collects the debris, effectively preventing damage to the net material.

Water jet technology encompasses rotating water jet cleaning, high-pressure water jet cleaning, and cavitation jet technology. Rotating jet-cleaning technology employs one or more high-speed rotating jet discs to generate water streams that cover the net coat in a fan or circular pattern under centrifugal force, enabling efficient cleaning. Baba [54] applied rotating jet-cleaning technology to a net-cleaning robot and integrated it with an image processing system to achieve precise cleaning of net coatings. Osaka [55] developed a screw-propelled underwater cleaning robot that effectively adapts to the net coating of a net and uses rotating jets to remove attached material. Reid [56] developed a high-pressure swivel joint assembly that links the fluid hose to the internal wash system, generating a high-pressure vortex that not only enhances the efficiency of the net-coating-cleaning process but also minimizes damage to the mesh material. Jichao Zhuang [57] and colleagues designed a floating net-cleaning robot that integrates high-pressure rotating water jet technology with a manifold spray disc to expand the cleaning coverage area of the net coating.

High-pressure water jet cleaning technology uses a high-speed water stream generated by a high-pressure pump to form an extremely fine jet through a nozzle, which directly impacts the dirt on the net coating, effectively achieving cleaning [58,59]. Building on this, Peter [60] integrated high-pressure water jet technology into an automated net-coating-cleaning robot, and integrated it with a manipulator to enhance the cleaning effect. In Japan, Niigata Engineering [61] developed the ‘Aqua Boy’ underwater robot, which uses high-pressure water jet technology and combines rotating brushes to effectively remove net attachments through the dual action of physical friction and water impact. Xiaoming Zhang [62] designed a manifold-type high-pressure jet underwater net washing machine, which can form a high-pressure jet through a high-pressure pump and nozzle to achieve the efficient cleaning of net coatings. This design optimizes the distribution and pressure of the water flow and improves the uniformity and efficiency of cleaning. Xiefa Song [63] combined high-pressure jet technology with brush-cleaning technology in their study and used hydrodynamic calculations based on Bernoulli’s equation and the momentum theorem to optimizes the design of the cleaning equipment to ensure that high-pressure water jets are efficient in practice.

Cavitation jet technology relies on the high speed of water flow through a cavitation nozzle, which generates a low-pressure zone that forms cavitation bubbles. When a large number of cavitation bubbles collapse and impact the localized net coating, a strong high-pressure micro-jet is produced, effectively cleaning the adhesion on the mesh coat [64]. Cavitation jet technology is not only effective in removing stubborn dirt from the net coating but also causes less damage, making it a more environmentally friendly and efficient cleaning method. Zhejiang University [65] has researched an underwater cleaning robot with an adaptive mechanism to address the stability and maneuverability of underwater cleaning robots. The robot adopts passive rolling joints and an optimized combined magnetic adsorption unit to ensure its stability and mobility in the variable marine environment. Meanwhile, the robot is equipped with cavitation jet technology to achieve the efficient cleaning of steel component surfaces.

In cavitation jet technology, the cavitation efficiency of the nozzle directly impacts the cleaning efficiency of the mesh coat. To uncover a more effective cavitation cleaning mechanism, Haitham [66] conducted a detailed analysis of the inter-hole interactions in the hydrodynamic cavitation process of a multi-hole nozzle (MHO). He found that optimizing the hole spacing can significantly enhance turbulence intensity and bubble generation efficiency. Hutli [67] investigated the dynamic characteristics of the cavitation cloud using high-speed photography and image processing techniques, revealing that increased injection pressure enhances both the penetration depth and the total area of the cavitation cloud. Kewen [68] investigated the cavitation phenomenon in water jets under high ambient pressure conditions through experiments and numerical simulations. They found that the bubble transport capacity in a cavitating jet is related to the jet velocity, and that the jet must have a sufficient flow rate to prevent bubble collapse inside the nozzle. Liang [69] demonstrated through numerical simulations in an AUV waterjet propulsion system that a cosine-shaped nozzle offers significant advantages in controlling cavitation and enhancing momentum-thrust conversion efficiency. Yang [70] found that the contraction degree and central body diameter of a novel central body nozzle are the most significant factors influencing cavitation. Their geometric parameter study revealed that when the contraction ratio and central body diameter reach optimal values, an effective low-pressure region is formed around the nozzle, promoting the generation of numerous bubbles and enhancing cavitation.

**Figure 7:**
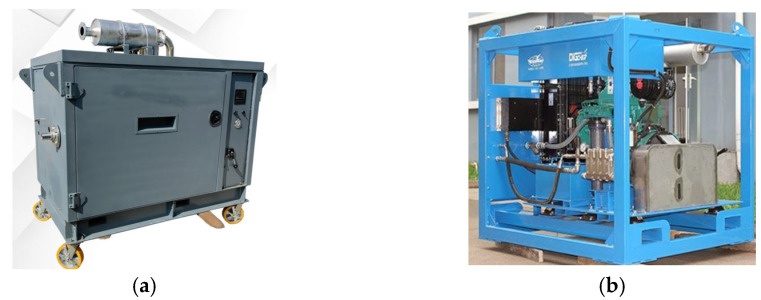
Cavitation jet external equipment for net-cleaning robots. (**a**) Sencott net-cleaning robot external cavitation jet high-pressure pumping unit. (**b**) External cavitation jet high-pressure pumping unit for DiGCHER net-washing machines.

As an advanced underwater cleaning technology, cavitation jet technology offers significant advantages in enhancing cleaning efficiency and minimizing damage to cleaned objects. However, it still faces several challenges in practical application. First, the external pressurization equipment is bulky, and the thick water jet transmission pipes may hinder the mobility and flexibility of the net-cleaning robot. Second, the noise generated by the pressurization equipment and the explosive effect of cavitation bubbles can disrupt the underwater ecosystem, potentially scaring or even killing fish. Furthermore, the actual cleaning results depend on factors such as equipment design, operating conditions, and environmental influences, meaning that cleaning efficiency may not always meet theoretical expectations. Figure 7 shows the cavitation jet external device of the net cleaning robot.

Currently, most nets are made from flexible or semi-flexible materials, providing the necessary support and permeability. However, these designs may experience a certain degree of physical wear and tear during the cleaning process with net-cleaning robots. Additionally, when the cleaning duration extends to 6–8 h or more, the energy consumption of water jet technology during such continuous operations becomes particularly significant. To reduce energy consumption and improve cleaning efficiency, future technological advancements will need to focus on two key areas: first, the development of more refined cleaning strategies to minimize the physical impact on the material of the nets; and second, the enhancement of energy efficiency and endurance in cleaning robots to ensure they can operate continuously without energy constraints.

### 3.4. Motion Control Technology for Net-Cleaning Robot

Net-cleaning robots are specifically designed for use in marine aquaculture environments. Unlike other underwater robots, their primary task is to operate within the net box, where they face challenges such as limited working space and the need to securely grip the net for effective operation. The control technology for net-cleaning robots can be divided into two categories: net inspection control and net adsorption control. Net box inspection control involves the robot autonomously inspecting the interior of the net box, assessing the condition of the net coat, and planning the cleaning path. Net box adsorption control requires the robot to remain in close proximity to the net coat during the cleaning process to ensure optimal cleaning performance. To meet these two control requirements, various control methods have been explored both domestically and internationally. These methods primarily include PID control, fuzzy control, and neural network control.

PID control is a classical feedback control method that achieves dynamic system regulation by adjusting three parameters: proportional (P), integral (I), and derivative (D). In net box inspection control, PID control can be used to adjust the robot’s speed and direction, enabling it to adapt to the complex environment inside the net box. In net box adsorption control, PID control helps the robot remain stable and maintain close proximity to the net coat, ensuring precise cleaning. However, the performance of PID control may be limited when applied to nonlinear systems, and it requires extensive experience in parameter tuning. In their study, Sarhadi [71] proposed a model-referenced adaptive PID control scheme. This scheme incorporates an adaptive anti-integral saturation (AW) compensator to enhance robustness against the actuator saturation problem.

The mathematical model of a net-cleaning robot is a typical nonlinear problem, and fuzzy control utilizes fuzzy logic to address uncertainty and nonlinear issues. In net box inspection control, fuzzy control can effectively handle the uncertainties encountered by the robot during the inspection process, such as the irregular shape of the net coat and the uneven distribution of dirt. However, designing and tuning fuzzy rules requires specialized knowledge, and predicting and analyzing control performance is challenging [72].

**Figure 8:**
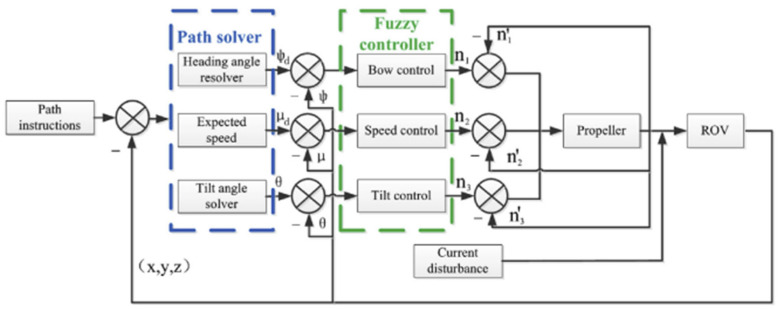
‘Sea Dog AUV’ fuzzy control system diagram [74].

To address these issues, Xiao Liang [73] proposed an enhanced fuzzy neural network control method that integrates a genetic algorithm with neural network self-learning to optimize the parameters of the affiliation function and dynamically adjust the fuzzy rules. Similarly, Chen [74] designed a fuzzy controller based on the line-of-sight method and further optimized it using a genetic algorithm. The effectiveness and robustness of this control method in complex marine environments were validated through simulations and experiments conducted on the ‘Sea Dog AUV’. Figure 8 is ‘Sea Dog AUV’ fuzzy control system diagram.

**Figure 9:**
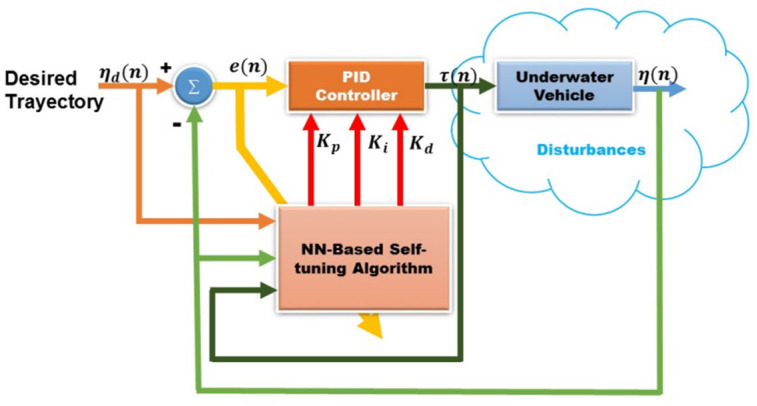
Block diagram of an auto-tuned PID with artificial NN control [76].

Neural network control can be achieved by learning all or part of a nonlinear system and training the neural network to control complex systems. It offers strong generalization ability and adaptability, with the key advantage that it does not require complete knowledge of the net-cleaning robot’s dynamics. In net adsorption control, the neural network can automatically adjust the robot’s adsorption strategy in response to changes in adsorption force, allowing it to adapt to different cleaning requirements. Currently, researchers are also exploring the application and improvement of neural network control methods, Yuh [75] designed a neural network controller and applied it to the control of URVs. Simulation results verified that neural network control can effectively deal with uncertainties and perturbations in complex environments. Hernández-Alvarado [76] proposed an adaptive PID controller based on neural networks, which utilizes a neural network to automatically adjust the PID parameters, ensuring efficient and precise control despite variations in system parameters or unknown disturbances. Figure 9 is Block diagram of an auto-tuned PID with artificial NN control. Mingjun Zhang [77] modeled and controlled an autonomous underwater vehicle (AUV) using a generalized predictive control method based on neural networks, incorporating an improved Elman network. The system’s adaptability to dynamic changes was significantly enhanced through an online learning mechanism.

With the rapid development of artificial intelligence technology, reinforcement learning control strategies have become a cutting-edge focus in the field of net box-cleaning robot control. Reinforcement learning adjusts the behavior of a net box-cleaning robot by receiving feedback (reward or punishment) from the environment, aiming to maximize long-term cumulative rewards. In the application of net box-cleaning robots, reinforcement learning not only enhances their ability to adapt to dynamic environments but also enables autonomous decision-making and learning in unknown or changing conditions.

Reinforcement learning is increasingly emerging as a key research focus in the control of net-cleaning robots, thanks to its strengths in handling unknown dynamics, uncertainty, and real-time adaptation. Elhaki [78] proposes an adaptive robust neural network control method that integrates reinforcement learning with dynamic surface control theory. Utilizing an Actor–Critic neural network architecture, this approach compensates for system dynamics, environmental disturbances, and actuator saturation. Furthermore, it enhances system responsiveness to unknown dynamics and strengthens robustness through real-time online training. Carlucho [79] developed an adaptive low-level control strategy based on deep reinforcement learning. This strategy employs a deterministic policy gradient algorithm and a deep neural network to approximate value functions and policies within a continuous action space, effectively addressing challenges in the bottom-level control of autonomous underwater vehicles (AUVs). Wang [80], on the other hand, proposed a robust deep tracking control method based on adversarial deep reinforcement learning, specifically designed to address the deep tracking challenges of underactuated autonomous underwater vehicles (AUVs) in the presence of complex dynamics and external disturbances.

**Figure 10:**
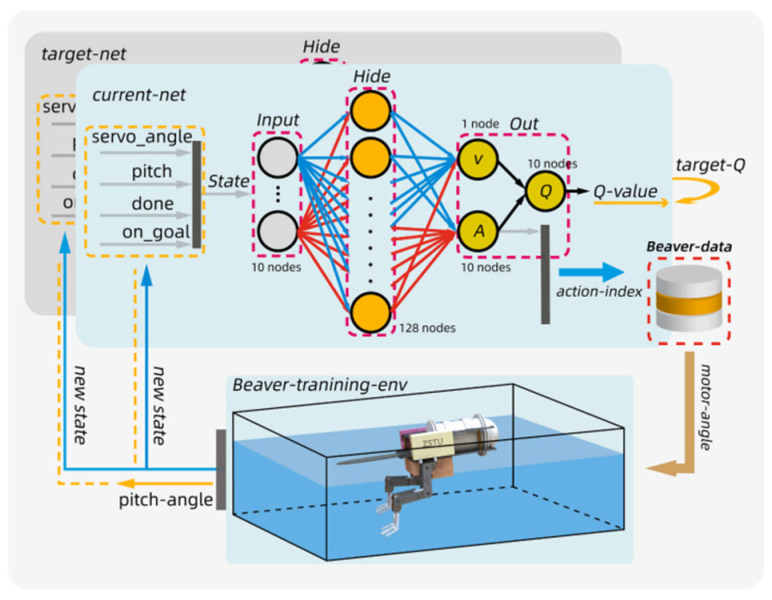
Pitch attitude stabilization control system of beaver-like underwater robot [81].

In applying these methods, reinforcement learning not only adaptively optimizes control strategies but also gradually mitigates the uncertainty caused by environmental changes through a continuous trial-and-error process. Chen [81] proposed a control algorithm based on deep reinforcement learning, which has been successfully applied to the attitude control of a beaver-inspired bionic robot. Utilizing the DRL algorithm, the robot achieves autonomous learning without requiring a complex higher-order model and optimizes its swimming strategy to maintain a stable pitching attitude. This study demonstrates that deep reinforcement learning can effectively control physical systems and manage complex control tasks in real-world applications without relying on a complete model. Figure 10 is pitch attitude stabilization control system of beaver-like underwater robot.

In addition to the control techniques mentioned above, the field of net-cleaning robot control has also explored methods such as sliding mode control, inverse control, and fault-tolerant control, each of which offers unique advantages and is capable of addressing specific challenges and requirements. However, in practical applications, a single control method may struggle to meet all requirements. As a result, the control technology of net-cleaning robots has gradually shifted towards composite control methods that integrate multiple strategies to achieve improved performance and greater adaptability.

### 3.5. Underwater Positioning Navigation and Motion-Planning Technology

When performing cleaning tasks underwater, net-cleaning robots rely on precise positioning, navigation, and efficient motion planning techniques to ensure the accuracy and safety of their operations. However, these technologies still face several challenges: (1) underwater uncertainty, which can affect the positioning accuracy and path planning of net-cleaning robots; (2) the nonlinear dynamics of six-degree-of-freedom underwater robots, which complicate the establishment of motion models; and (3) the complexity of sensor data fusion in turbid environments, along with the need to adapt to dynamic obstacle avoidance due to variable net box structures.

#### 3.5.1. Underwater Positioning and Navigation Technology for Net-Cleaning Robots

Underwater positioning and navigation technology is critical to the success of net-cleaning robots. Currently, underwater positioning technologies primarily include acoustic, visual, laser, and inertial navigation systems. Acoustic positioning is the most mature and widely used method for underwater robot positioning and navigation. It relies on the propagation characteristics of sound waves in water to determine the robot’s position by transmitting and receiving acoustic signals. Acoustic systems include long baseline (LBL), short baseline (SBL), and ultra-short baseline (USBL) positioning and navigation systems. Visual navigation techniques rely on image data captured by underwater cameras, but visibility is significantly reduced in turbid waters, leading to lower positioning accuracy. Underwater LiDAR (LIDAR) is an emerging method of underwater navigation. LiDAR calculates the distance between the underwater robot and surrounding objects by emitting laser pulses and measuring the time it takes for them to return after reflecting off the objects. It maintains strong performance even in low-visibility conditions. However, LiDAR systems are relatively expensive and sensitive to reflections and scattering from the water surface. Inertial navigation systems, on the other hand, determine the robot’s position and attitude by measuring acceleration and angular velocity, but they accumulate errors over time. To improve the positioning and navigation accuracy of net-cleaning robots, Yuliang Xu [82] proposed an integrated approach. This approach employs a multi-sensor data fusion method to estimate the robot’s velocity and position, combined with filtering algorithms to optimize sensor data processing, achieving accurate navigation and control of the robot’s six-degree-of-freedom motion.

A single navigation technique is often insufficient to meet the demands of high-precision positioning and navigation. Combining multiple navigation techniques allows them to complement each other, improving both accuracy and robustness. For example, acoustic positioning can provide coarse positioning over a wide area, which can then be refined through visual or laser navigation.

#### 3.5.2. Motion Planning for Net-Cleaning Robots

Path-planning techniques for net-cleaning robots ensure both efficiency and safety in task execution. Due to the unique characteristics of the workspace, there are almost no obstacles on the net surface. Compared with other types of underwater robots, the path planning for net-cleaning robots typically emphasizes efficiency and coverage rather than obstacle avoidance. The path planning of net-cleaning robots can be broadly categorized into global path planning and local path planning. Common path-planning methods include graph search algorithms, artificial potential field (APF), random sampling methods, and artificial intelligence algorithms. Table 4 systematically summarizes and compares various common path planning algorithms.

In artificial intelligence-based path planning methods, reinforcement learning algorithms can be applied to enable robots to interactively learn from the environment and adjust their strategies based on reward signals from their behaviors. This allows net-cleaning robots to maximize long-term rewards in dynamic path planning scenarios [102]. Liu [103] proposed a reinforcement learning-based path planning method for autonomous underwater vehicles (AUVs), effectively addressing uncertainty and variability in dynamic marine environments, and particularly achieving optimal path planning under complex current conditions. The innovation of this method lies in its reinforcement learning algorithm, which autonomously adjusts its strategy to cope with dynamic changes and uncertainties in complex environments. Similarly, Cui [104] proposed an adaptive neural network control method to address the trajectory tracking problem of autonomous underwater vehicles (AUVs) under control input nonlinearity and model uncertainty, using reinforcement learning techniques. This method effectively handles model uncertainty and complex control challenges by adaptively adjusting the control strategy, thereby improving the trajectory tracking accuracy of the AUV.

The deep reinforcement learning algorithm integrates the feature extraction capability of deep learning to approximate complex value functions or policy functions by training deep neural networks. This approach is particularly well-suited for addressing the challenges of underwater, multi-dimensional environments with continuous action spaces, such as net cleaning. Pengcheng Fang [105] proposed an improved Dueling DQN algorithm for path planning in three-dimensional marine environments, factoring in the influence of ocean currents to enhance the real-time performance and accuracy of AUV path planning. Similarly, Yu [106] proposed an optimal trajectory tracking control method based on deep reinforcement learning to precisely control the trajectory tracking of an autonomous underwater vehicle (AUV) using two neural networks and the deep deterministic policy gradient (DDPG) algorithm. Compared with traditional methods, the DDPG algorithm is better equipped to handle nonlinear control problems in continuous action spaces, achieving accurate trajectory tracking, especially in complex and uncertain marine environments, demonstrating strong robustness.

**Figure 11:**
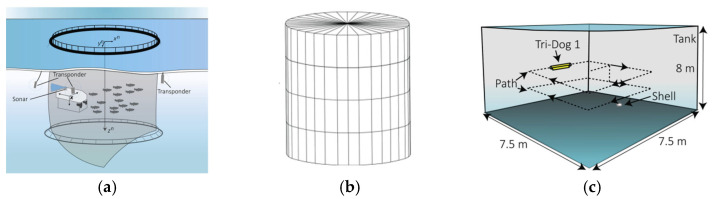
Environmental mapping of net-pen aquaculture. (**a**) structured; (**b**) discrete; (**c**) Cruise Route Preset [107].

The effective implementation of various net-cleaning path-planning methods depends on the construction of a structured fishery environment. A structured environment not only provides the necessary operational space for the net-cleaning robot but also offers a standardized layout that can significantly simplify the complexity of path planning. Structuring the spatial environment of net-tank farming, followed by discrete partitioning to create a water body map, enhances the efficiency of net-tank cleaning. Using the target work area to guide the robot’s path further optimizes the process [107]. This structured design helps the net-cleaning robot better understand and predict its working environment, thereby improving its autonomy and adaptability in real-world applications. Figure 11 is environmental mapping of net-pen aquaculture.

A unique challenge in map construction for net-cleaning robots is that the workspace primarily focuses on the net surface, which dynamically changes under the influence of current loads. Therefore, map construction must not only capture the location and structure of the net but also adapt to and predict its dynamic changes. To address this, net map construction can be integrated with hydrodynamic modeling, machine vision, and SLAM technology. Hydrodynamic modeling simulates the net’s dynamic response to water flow, updating its position and shape in real time. Machine vision monitors the net’s condition in real time, identifying dirt and damage and incorporating this data into the map. SLAM technology assists the net-cleaning robot in autonomously localizing itself in unknown environments while simultaneously constructing the environment map.

The ability of a net-cleaning robot to follow its planned motion is primarily constrained by two factors: the accuracy of its dynamics model and external environmental disturbances. The accuracy of the dynamics model affects how closely the actual motion aligns with the planned motion. Therefore, incorporating the kinetic model into motion planning as a regular constraint, with a combination of strong and weak constraints, helps enhance the robustness of the path planning. Additionally, when the robot cleans the net, the net surface inevitably fluctuates due to ocean currents or wave loads, impacting the accuracy of the path. As a result, these external factors must be carefully considered in the planning process to achieve optimal path planning for the net-cleaning robot.

Path planning for net-cleaning robots is a complex problem that involves multi-dimensional and multi-technology integration. Ranging from classical graph search algorithms to modern artificial intelligence methods, each technology offers unique advantages and applicable scenarios. As technology continues to advance, the future path planning for net-cleaning robots will become increasingly intelligent and automated, enabling them to better address various challenges and task requirements.

## 4. Conclusions and Prospects

Net cleaning represents a typical ‘3D’ (boring, repetitive, and fine) mode of work that demands a high degree of patience and technical skill. The introduction of net-cleaning robots into the aquaculture industry not only simplifies net cleaning but also reduces labor costs and operational risks by reducing manual intervention, while improving cleaning consistency and efficiency.

Net-cleaning robots can operate in complex and dynamic underwater environments, minimizing the risk of direct human contact with hazardous conditions. However, the intricacy of these underwater environments can directly impact the robot’s control performance and cleaning effectiveness. Additionally, factors such as wave loads and marine organisms can reduce the accuracy of the net-cleaning robot’s perception of external information. Furthermore, underwater signals may be attenuated and interfered with, affecting real-time communication between the robot and the host computer.

In response to the complexities of underwater environments, research in net-cleaning robotics is focusing on key technological breakthroughs to advance automation technologies. This includes the following:

(1) Intelligent Underwater Vision and Communication Technology: An advanced underwater vision system must integrate high-performance optical and acoustic imaging equipment while also incorporating deep-learning and image-processing algorithms to achieve high-precision identification and classification of net box structures, attached organisms, dirt, and other targets. Additionally, to ensure real-time data exchange between the net-cleaning robot and the control center, underwater communication technology must integrate adaptive modulation and multi-path transmission technologies to enhance communication efficiency in complex underwater environments.
(2) Adaptive Net-Fitting Motion Technology: Net-fitting motion is a fundamental capability that a net-cleaning robot must possess. The tracked motion mechanism provides stable movement and allows the robot to adhere securely to the net, while the adaptive gripping hook ensures a close attachment between the robot and the net surface. Additionally, the screw propeller can adjust the robot’s attitude while generating significant propulsive force. To effectively handle various net structures and dynamic water flow conditions, future research should incorporate multimodal sensor fusion technologies (e.g., force, vision, and haptics), enabling the net-cleaning robot to accurately sense and respond to the state of the net surface, thereby achieving highly efficient and precise net-cleaning movements.
(3) Innovative Low-Energy Cleaning Methods: Low-energy cleaning methods should prioritize the balance between cleaning efficiency and energy consumption while minimizing damage to the mesh coat. Future developments will integrate lightweight scraping, bubbling, low-pressure water flow, and water jet technology. In particular, while cavitation jet technology is effective in dirt removal and reducing damage to the mesh coat, its high energy consumption remains a challenge. This can be addressed by optimizing nozzle design and adjusting the bubble generation mechanism to reduce energy consumption, as well as by innovatively designing a new low-power cleaning solution that combines multiple scraping and brushing techniques.
(4) Motion Coordination Control Technology: Net-cleaning robots must operate with multiple degrees of freedom, necessitating a high level of coordination and precision in their motion control systems. Future technological advancements will focus on enhancing the intelligence of these control systems to manage complex movements and adapt to changing environmental conditions with greater accuracy. Additionally, the development of multi-robot collaboration technologies will enhance operational efficiency and adaptive capabilities through collective intelligence and communication protocols.
(5) Artificial Intelligence-Based Positioning and Planning Technology: The underwater environment cannot be directly positioned using GPS, so the net-cleaning robot must rely on the fusion of acoustic positioning, visual inertial navigation, and simultaneous localization and mapping (SLAM) technologies to achieve accurate positioning. In terms of path planning, the integration of AI technology has become an inevitable trend. By applying AI technologies such as Deep Reinforcement Learning (DRL), the net-cleaning robot can autonomously learn and optimize its cleaning path during continuous operation, thereby improving cleaning efficiency.
(6) Biological Growth Characteristics and Cleaning Strategies: The species and densities of attached organisms vary significantly across different waters and net materials. The integration of biological growth characteristics and cleaning strategies can greatly enhance cleaning effectiveness while minimizing damage to the structure of the nets. In the future, utilizing big data analysis and machine-learning algorithms to analyze the spatial and temporal distribution patterns of attached organisms will provide a scientific basis for optimizing cleaning frequency and methods. Additionally, the development of new cleaning tools and biological inhibitors, combined with the adjustment of intelligent cleaning strategies, will enable the net-cleaning robot to minimize damage to the nets and ensure the efficiency and sustainability of the cleaning operation.

In addition, the research and practical application of net-cleaning robots still lacks studies on cleaning efficiency, adaptive net adhesion force technology, digital twin dynamic modeling of the net coat under real conditions, and AI-based big data decision-making technology. As technology and equipment continue to evolve, these technical areas will also be further explored and refined in the future.

In summary, the net-cleaning robot is key equipment for the transformation and upgrading of intelligent operation and maintenance in marine fisheries. However, in current practical applications, it is constrained by existing technologies and cannot yet perform net-cleaning tasks completely independently, efficiently, or conveniently. Looking ahead, fishery development should focus on integrating human expertise with the decision-making systems of net-cleaning robots. The use of big data technology and optimization algorithms will provide real-time feedback on optimal cleaning methods and paths. Ultimately, the goal is to achieve intelligent fisheries through human–machine collaboration.

## Figures and Tables

**Table 1:** Research Progress of Underwater Optical Recognition and Acoustic Recognition Technology.

Technology Category	Application Scenario	Researcher	Technical Characteristics	Problems or Adjustments Addressed
**Optical Recognition Technology**	**Underwater Target Identification**	Forst [24]	Identify and collect fish data using high-definition cameras	Automatic recognition under the influence of complex underwater environments
	**Automated Detection of Mesh Coat Breakage in Offshore Nets**	Jinquan Zhang [25]	The AUV comes with a video system and 3-degree-of-freedom controls	Detection of breakage of net clothing in offshore nets
	**Underwater Image enhancement**	Petit [26]	Quaternion Decay Inversion Method	Distorted images of underwater dirt
		Liang [27]	Backscattered light estimation with multilevel search	Estimation of underwater image transmission
		Liu [28]	Cascading Multi-Module Image Enhancement Framework	Media degradation and color distortion
	**Deep Learning**	Han [29]	Intelligent defogging and color restoration algorithms	Underwater image defogging and color recovery
		Zhou [30]	Multi-view underwater image enhancement method	Color deviation and blurred details
		Chen [33]	Deep learning combined with physical prior methods	Insufficient training data
		Siqi Lu [31]	A two-stage underwater image enhancement method	Color distortion and clarity issues
		Yuzhen Liu [32]	Multi-scale feature fusion and attention networks	Image detail enhancement and information suppression
**Acoustic Recognition Technology**	**Sonar Image Denoising**	Wang [34]	Sonar systems, acoustic propagation analysis	Sonar image noise suppression
		Li [35]	Sonar signal processing	Sonar image noise removal
		Cheng [36]	Self-supervised denoising technique with dynamic attention mechanism	Adaptation of sonar image characteristics

**Table 2:** Comparison of movement methods of net-cleaning robots.

Mode of Movement	Specificities	Advantage	Disadvantage	Reference
**Wheeled**	Direct motor-driven wheel movement, can be fitted with gimbals	Flexible motion and fast response time	Low friction, poor barrier-crossing ability	[40]
**Foot-Style**	Relying on the soles of the feet to make contact with the surface of the net	Flexibility of movement and ability to cross obstacles	Slow, inefficient, poor load capacity	[41]
**Caterpillar Track**	By clicking to turn the track, the track moves in contact with the mesh coat	High friction and high barrier-crossing ability	Steering is prone to skidding	[39]

**Table 3:** Comparison of the characteristics and applications of mesh coat decontamination technology.

Type of Technology	Specificities	Applicable Occasions	Advantage	Disadvantage	Reference
**Lightweight Scratch and Flush Technology**	Integrated scraping and low-pressure flushing for low energy consumption	Surface cleaning of mesh clothing	Low energy consumption, no damage to mesh material	Average cleaning efficiency	[52,53]
**Rotary Water Jet Cleaning**	Using a high-speed rotating jet disc to generate a stream of water that covers the mesh coat for cleaning	Occasions where the surface of the mesh coat is heavily soiled and requires extensive cleaning.	High cleaning efficiency and wide coverage	May cause some damage to the mesh coat, high energy consumption	[54,55,56,57]
**High Pressure Water Jet Cleaning**	The high-speed water flow generated by the high-pressure pump is used to create a fine jet through the nozzle to clean the mesh coat	Where the surface of the mesh garment is stubborn and needs to be power washed	Strong cleaning power to remove stubborn dirt	May cause some damage to the mesh coat, high energy consumption	[58,59,60,61,62,63]
**Cavitation Jet Technology**	Through the cavitation nozzle to produce a low-pressure area to form cavitation bubbles, the use of bubble collapse generated by the high-pressure micro-jet cleaning	When there is stubborn surface dirt on the mesh and the goal is to minimize damage to the mesh.	Good cleaning effect, little damage to the mesh coat, environmentally friendly	Complex technology, higher equipment costs, higher energy consumption	[64,65]

**Table 4:** Underwater Path Planning Algorithm.

Route Planning Methodology	Concrete Method	Peculiarity	Applicable Environment	Advantage	Limitations	Reference
**Graph Search Method**	**Dijkstra**	Step-by-step updating of distance information to find the shortest path	Static, no-negative-weight edges environment	(1) Accurate calculation of the shortest paths in static underwater environments. (2) Applicable to underwater maps without negative weights. (3) Algorithm is robust and easy to implement.	(1) Calculation volume increases significantly with the size of the underwater environment, unsuitable for large-scale planning. (2) Inability to cope with dynamic obstacles or environmental changes.	[83]
	A*	Heuristic search to optimize path planning for static environments	Static environment	(1) Combine with heuristic search to improve efficiency, suitable for underwater path optimization. (2) Provide higher accuracy planning results in known static environments.	(1) Heuristics need to be optimized for underwater environment characteristics. (2) Response to dynamic obstacles and environmental changes is slow.	[84,85]
	D*	Optimization of the A* algorithm	Dynamic environment	(1) Designed for dynamic environments, can dynamically adjust the underwater route. (2) Adaptability to changes in the environment such as water currents and obstacles.	(1) Complexity of computation is high, not suitable for tasks with very high real-time requirements. (2) May perform poorly in extremely complex dynamic underwater environments.	[84,86]
**Artificial Potential Field Method**	**APF**	Attractive potential field guidance, repulsive potential field avoidance	Static environment	(1) Intuitive and simple, can quickly plan paths and avoid obstacles. (2) Applicable to local planning or real-time obstacle avoidance tasks, such as underwater robot proximity navigation.	(1) Prone to local minima, resulting in the inability to find a globally optimal path. (2) Limit performance in complex underwater terrain, such as narrow areas or multi-obstacle scenarios.	[87,88,89]
**Random Sampling Method**	**RRT**	Random sampling to explore the state space	High-dimensional, dynamic environment	(1) Suitable for complex or high-dimensional underwater environments, can quickly generate feasible paths. (2) Poor environmental modelling requirements and high adaptability.	(1) Path is usually not smooth or optimal, requires post-processing optimization. (2) response is slow in highly dynamic underwater environments.	[90,91]
	**PRM**	Random sampling to generate waypoints and build graphical networks	Static environment	(1) Suitable for global path planning in static underwater environments, can quickly find feasible paths. (2) Performs well in a wide range of waters.	(1) Route quality is dependent on sampling density and may not be optimal. (2) Difficulty in adapting to dynamic obstacles or real-time changes in the environment.	[92,93]
**Artificial Intelligence Algorithm**	**GA**	Simulating Genetic Evolution to Search for Optimal Paths	Dynamic and complex environment	(1) Capable of global path planning for complex underwater environments and able to handle multi-objective optimization. (2) Objective function sensitive, can be adjusted with task characteristics.	(1) Computationally expensive, difficult to meet real-time requirements. (2) Convergence is slow and performance is limited in dynamic environments.	[94,95]
	**ACO**	Simulating ant behavior to search for optimal paths	Dynamic environment	(1) Adaptive and able to dynamically respond to changes in the underwater environment. (2) Available for global path optimization, especially effective in complex underwater networks.	(1) Requirements for modelling environmental features are high and parameter tuning is complicated. (2) Convergence is slow in sparse or discontinuous environments.	[96,97]
	**PSO**	Simulating group behavior to find the optimal path	Continuous space	(1) Applicable to underwater continuous path optimization with high computational efficiency. (2) Utilizing group collaboration, easy to achieve multi-objective optimization.	(1) Adaptation to discrete environments is poor. (2) Potential to fall into local optima in complex obstacle environments.	[98,99]
**Machine Learning Methods**	**NN**	Mimic brain neuron	High dimension	(1) Apply to path learning and planning in complex underwater scenes. (2) Integrated with underwater sensor data to improve planning intelligence.	(1) Requires large amount of underwater scene data for training. (2) The model has limited ability to generalize to unknown scenes.	[100,101]
	**RL**	Reward Signal Adjustment Strategies	Dynamic, uncertain environment	(1) Able to adapt and adjust autonomously in dynamic, uncertain underwater environments. (2) Requires no precise environmental modelling and is suitable for unstructured scenarios.	(1) Training requires large amounts of interaction data and is costly. (2) Learning process is highly dependent on feedback from complex dynamic environments.	[102,103,104]
	**DRL**	Combining Deep Learning and Reinforcement Learning	High dimensional complex environment	(1) Combining perception and control for high-dimensional underwater dynamic environments. (2) Handling complex non-linear underwater path planning tasks.	(1) Algorithm complexity is high and requires a lot of computational resources. (2) Training time is long and difficult to apply directly to real-time planning.	[105,106]

## Data Availability

Not applicable.
